# Histopathology and enhanced detection of tumor invasion of peritoneal membranes

**DOI:** 10.1371/journal.pone.0173833

**Published:** 2017-03-10

**Authors:** Jey-Hsin Chen, Melissa Borges

**Affiliations:** 1 CellNetix Pathology and Laboratories, Seattle, Washington, United States of America; 2 Swedish Medical Center, Seattle, Washington, United States of America; Hospital Universitario de la Princesa, SPAIN

## Abstract

Tumor invasion of the peritoneal membrane may have an adverse prognostic significance, but its histopathologic features can be diagnostically difficult to recognize. We observed that local peritoneal injury associated with tumor invasion is characterized by activation and proliferation of serosal stromal cells that express cytokeratin, a characteristic property of injured serosal membranes that may have diagnostic utility. To explore this, we examined 120 primary tumors of the gastrointestinal tract and pancreaticobiliary system using cytokeratin and elastic stains to assess for tumor invasion of peritoneal membranes. Peritoneal invasion by tumor was associated with retraction, splaying, and destruction of the elastic lamina and proliferation of keratin-expressing stromal cells of serosal membranes. All 82 peritoneal invasive tumors were characterized by neoplastic cells that invaded the elastic lamina and the serosal connective tissue with neoplastic cells that abutted or were surrounded by keratin-positive stromal cells, whereas all 38 tumors limited to the subserosa showed none of these features. The diagnosis of tumor invasion of peritoneal membranes is enhanced by the combined use of cytokeratin and elastic stains, which in turn would enable better histopathologic correlation with patient treatment and outcome.

## Introduction

The prognosis and multidisciplinary treatment of tumors of the gastrointestinal and pancreaticobiliary system are guided by tumor staging classifications such as the American Joint Committee on Cancer (AJCC) and International Union Against Cancer (UICC) [1,2]. In these classifications, the depth of mural invasion is correlated with the pathologic tumor stage (pT). Complete mural invasion with tumor penetration of the peritoneal surface is a well-recognized adverse prognostic feature that is associated with an increased risk for tumor spread and recurrence, particularly within the peritoneal cavity, and this is reflected by a high pathologic tumor stage [3–8]. The clinical significance of tumors that invade the peritoneal membrane without serosal surface involvement, however, is less clear [9–14]. Recognizing tumor invasion of peritoneal membranes can be diagnostically challenging. Invasion of the elastic lamina characterizes tumor invasion of the peritoneal membrane, but the elastic lamina can be difficult to detect due to its displacement, splaying, and fragmentation when invaded by tumor [9,11,12].

Injured peritoneal membranes are associated with activation and proliferation of immature serosal stromal cells that express low molecular weight cytokeratin, a unique feature of serosal membranes that is not observed in stromal cells of extraperitoneal tissue [15–17]. Because the peritoneum is locally injured at or near the site of deep tumor invasion, we reasoned that the expression of cytokeratin in serosal stromal cells may aid in the diagnosis of tumor invasion of peritoneal membranes. To investigate this, we examined the histopathologic changes in peritoneal membranes, and assessed the practical diagnostic utility of cytokeratin immunohistochemistry in combination with an elastic stain in the diagnosis of tumor invasion of peritoneal membranes in a variety of deeply invasive tumors of the gastrointestinal tract and pancreaticobiliary system.

## Materials and methods

The study was reviewed and approved by the Institutional Review Board of Swedish Medical Center. Surgical resection specimens of 120 primary tumors of the colon and proximal rectum (56 cases), appendix (13 cases), small intestine (9 cases), distal esophagus and stomach (18 cases), pancreas (14 cases), and gallbladder (10 cases) that invaded the subserosa or peritoneal membrane were selected from the pathology archives of Swedish Medical Center and CellNetix Pathology and Laboratories for further analysis (Table 1 and S1 Table). Among the various tumors studied were adenocarcinoma and variants including poorly cohesive/signet ring cell carcinoma, colloid carcinoma, medullary carcinoma, and adenosquamous carcinoma (104 cases), neuroendocrine neoplasms including carcinoid tumors of the small intestine and adenocarcinoma ex goblet cell carcinoid of the appendix (13 cases), and low-grade appendiceal mucinous neoplasms (3 cases). The peritoneum from 3 to 5 cases of non-neoplastic stomach, small intestine, colon, proximal rectum, appendix, pancreas, and gallbladder were similarly examined in parallel. The tissues were fixed in 10% neutral buffered formalin and processed and stained with routine hematoxylin and eosin (H&E) and Verhoeff-Van Gieson (VVG). Immunostains for pan-keratin (46.3 μg/mL, Roche Ventana Medical Systems, Tucson, AZ, catalog number 760–2595) were examined following antigen retrieval and visualized using a polymer-based immunoperoxidase reaction and counterstained with hematoxylin. A small number of cases were additionally stained for CK7 (1:1200, Agilent Technologies, Santa Clara, CA, catalog number M7018), CK20 (1:400, Agilent Technologies, Santa Clara, CA, catalog number M7019), calretinin (1:20, Leica Biosystems, Buffalo Grove, IL, catalog number NCL-L-CALRET-566), WT-1 (1:100, Agilent Technologies, Santa Clara, CA, catalog number M3561), D2-40 (1:50, Biocare Medical, Concord, CA, catalog number 266), SMA (1:400, Agilent Technologies, Santa Clara, CA, catalog number M0851), and EMA (1:100, Agilent Technologies, Santa Clara, CA, catalog number M0613). Tumor invasion of the peritoneal membrane was defined by neoplastic cells that invaded the elastic lamina and the connective tissue of the serosal membrane, whereas tumors confined to the subserosa lacked neither invasion of the elastic lamina nor the peritoneal connective tissue (Table 1). Statistical significance was determined by Fisher’s exact test using R version 3.1.1 (R Foundation for Statistical Computing, Vienna, Austria, URL http://www.R-project.org).

**Table 1:** Tumor location, type, and depth of invasion.

	Tumor without invasion of the peritoneal membrane (i.e., confined to subserosa)	Tumor with invasion of the peritoneal membrane
**Colon and proximal rectum (N = 56)**		
• Adenocarcinoma (including 1 colloid carcinoma, 1 adenosquamous carcinoma, 1 medullary carcinoma)	18	36
• Poorly differentiated neuroendocrine carcinoma	1	1
**Appendix (N = 13)**		
• Adenocarcinoma (including 3 colloid carcinoma)	0	5
• Well-differentiated neuroendocrine neoplasm	0	2
• Adenocarcinoma ex goblet cell carcinoid	2	1
• Low-grade appendiceal mucinous neoplasm	0	3
**Small intestine (N = 9)**		
• Adenocarcinoma	1	2
• Well-differentiated neuroendocrine neoplasm	3	3
**Stomach (gastric or esophageal primary) (N = 18)**		
• Adenocarcinoma (including 5 poorly cohesive/signet ring cell carcinoma)	5	13
**Pancreas (N = 14)**		
• Ductal adenocarcinoma	5	9
**Gallbladder (N = 10)**		
• Adenocarcinoma (including 2 adenosquamous carcinoma)	3	7

## Results

**Fig 1:**
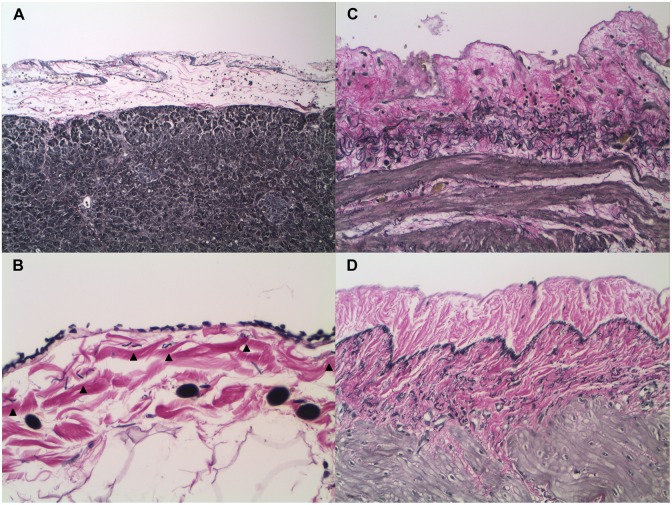
Variation in the light microscopic appearance of the peritoneal elastic lamina (VVG). The peritoneal elastic lamina, located between the mesothelium and the soft tissue of the subserosa and mesentery, is composed of a meshwork of elastic fibers and fibrils that typically appears thin and continuous by light microscopy (A, pancreas, 100x). Its appearance, however, can range from wispy, delicate, and discontinuous (B, appendix, 400x), to thick, ropy, and variably compact in long segments (C, stomach, 200x). In serosa that abuts near the muscularis propria, the elastic lamina appears to ramify with the elastic fibers of the subserosa (D, small intestine, 200x). Arrowheads: elastic lamina.

The elastic lamina of normal peritoneum is a reticular meshwork of interwoven elastic fibers and fibrils that appeared variably thick by light microscopy (Fig 1). It is typically thin and continuous (Fig 1A), but can range from barely discernable, wispy, and apparently discontinuous (Fig 1B), to thick, ropy, and continuous in long segments (Fig 1C). It is located almost immediately under the mesothelium and overlying the adipose tissue of the subserosa and mesentery. In areas in which the serosa abutted close to the muscularis propria, the elastic lamina appeared to ramify with the elastic fibers of the subserosa (Fig 1D).

**Fig 2:**
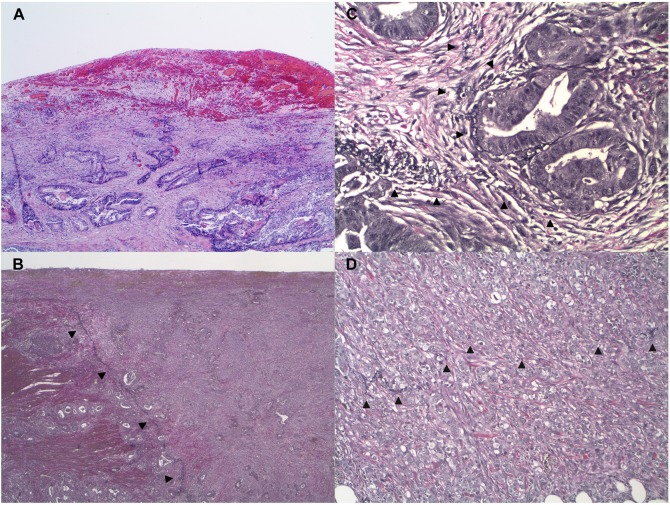
Histopathologic alteration of peritoneal membranes associated with tumor invasion (H&E and VVG). Tumor invasion of the peritoneal membrane is associated with localized inflammatory and desmoplastic reaction, resulting in hemorrhage and granulation tissue-like formation, surface fibrinous exudate, fibrosis, edema, and activation and proliferation of myofibroblast-like stromal cells (A, adenocarcinoma, colon, 40x). This is accompanied by retraction of the elastic lamina from the serosal surface toward the tumor (B, adenocarcinoma, colon, 20x) with splaying (C, adenocarcinoma, colon, 200x), attenuation, and fragmentation of its elastic fibers (D, adenocarcinoma, small intestine, 200x). Arrowheads: elastic lamina.

To characterize the histopathologic features associated with tumor invasion of the subserosa and peritoneum, we examined 120 primary tumors of the gastrointestinal tract, pancreas, and gallbladder (Table 1). Of these tumors, 82 invaded the peritoneal membrane to a variable degree, ranging from focal invasion of the elastic lamina and peritoneal connective tissue (i.e., partial or incomplete invasion of peritoneal membrane), to complete penetration of the peritoneal membrane with extension to the serosal surface (i.e., complete invasion of peritoneal membrane) (Fig 2). The peritoneal membrane is typically devoid of fat, in contrast to the subserosa and mesentery, and tumors that invaded the peritoneum invariably showed an absence of adipose tissue between the neoplastic cells at the point of deepest invasion and the serosal surface (Fig 2A). Deeply invasive tumors were often associated with a prominent inflammatory and desmoplastic reaction, resulting in retraction of the elastic lamina toward the tumor, marked in some instances with displacement of greater than 1 cm from the serosal surface (Fig 2B). There was variable splaying (Fig 2C), attenuation, fragmentation, and loss of the elastic fibers of the elastic lamina (Fig 2D). In cases in which the elastic lamina was difficult to detect due to tumor-associated retraction and destruction, it could be readily identified in areas away from the tumor between the serosal surface and the subserosal adipose tissue and then traced back toward the neoplasm.

**Fig 3:**
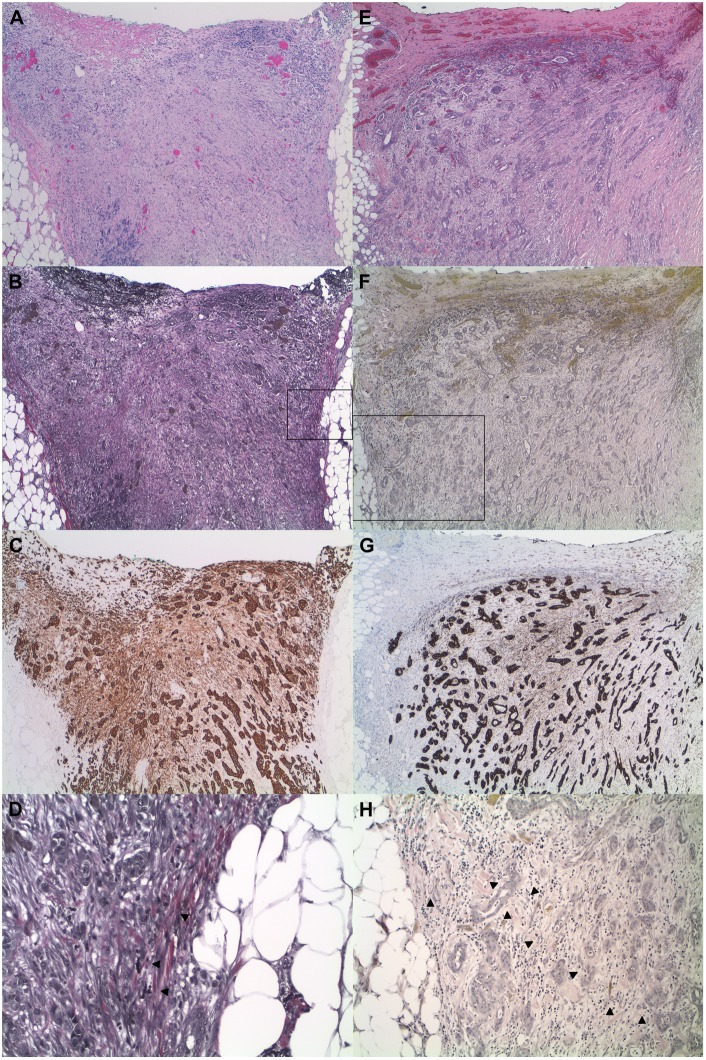
Tumor invasion of the peritoneal membrane is characterized by neoplastic cells that invade the elastic lamina and the peritoneal connective tissue with activation and proliferation of serosal stromal cells that express cytokeratin (H&E, VVG, and pan-keratin). Tumor invasion of the peritoneal membrane is associated with retraction, attenuation, and fragmentation of the elastic lamina and proliferation of serosal stromal cells that express cytokeratin, a unique feature not observed in stromal cells of extraperitoneal tissue. The keratin-positive stromal cells involve nearly the entire thickness of the expanded peritoneal membrane, forming a well-circumscribed population that is sharply delimited from the keratin-negative stromal cells of the subserosa by the elastic lamina. Neoplastic cells that invade the peritoneum abut or are surrounded by keratin-expressing stromal cells of the serosal membrane. (A-D, adenocarcinoma, small intestine, 40x, with higher magnification of boxed area in D, 200x; E-H, ductal adenocarcinoma, pancreas, 40x, with higher magnification of boxed area in H, 100x). Arrowheads: elastic lamina.

**Fig 4:**
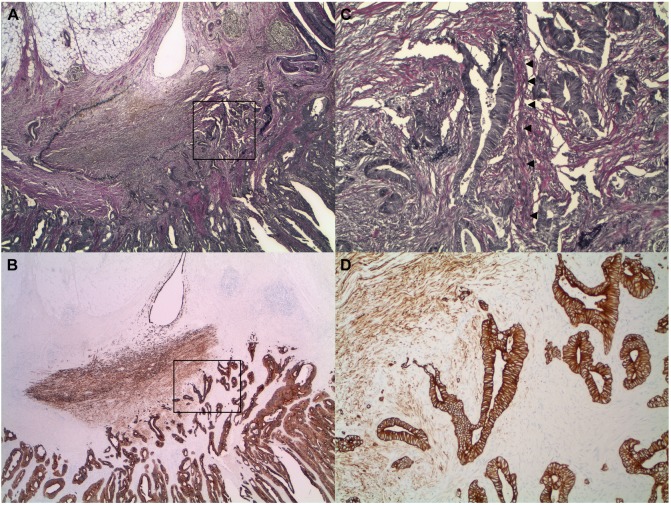
Keratin-positive stromal cells are confined within the elastic lamina of the peritoneal membrane (VVG and pan-keratin). Adenocarcinoma of the colon invades the peritoneal membrane with uneven fraying of the elastic fibers of the elastic lamina. At low magnification, the keratin-positive stromal cells appear to extend past the elastic lamina and into the subserosa (A and B, 20x). Closer inspection at higher magnification, however, reveals that the elastic lamina is unevenly splayed, with inner thin wispy fibers displaced from the bulk of the elastic lamina with deeper retraction toward the tumor (C and D, higher magnification of boxed areas, 100x). The keratin-positive stromal cells extend into the splayed elastic lamina, but are bounded by its innermost elastic fibers of the elastic lamina. Arrowheads: inner retracted elastic fibers of the elastic lamina.

Peritoneal injury was also characterized by expansion of the serosal connective tissue with hemorrhage and granulation tissue-like formation, fibrinous exudate at the serosal surface, fibrosis, edema, and activation and proliferation of myofibroblast-like stromal cells that expressed cytokeratin (Fig 3). This latter feature was specific to stromal cells of serosal membranes as there was no detectable keratin expression in stromal cells of extraperitoneal tissue. In most cases, keratin-positive stromal cells involved nearly the entire thickness of the peritoneal membrane and showed a gradient in the strength of keratin expression, with weaker reactivity near the elastic lamina and stronger expression toward the peritoneal surface (S1 Fig). This population was well-circumscribed and sharply delimited from the keratin-negative stromal cells of the subserosa by the elastic lamina. In fact, in diagnostically challenging cases in which the elastic lamina was difficult to identify, its location could be gleaned from the area of transition between the keratin-positive and keratin-negative stromal cells of peritoneal and extraperitoneal tissues, respectively. In some tumors that invaded a thick elastic lamina, there was fraying of the elastic lamina with unraveling of its elastic fibers. In these instances, the keratin-positive stromal cells extended into the splayed elastic lamina, but were bounded by its innermost elastic fibers (Fig 4).

**Fig 5:**
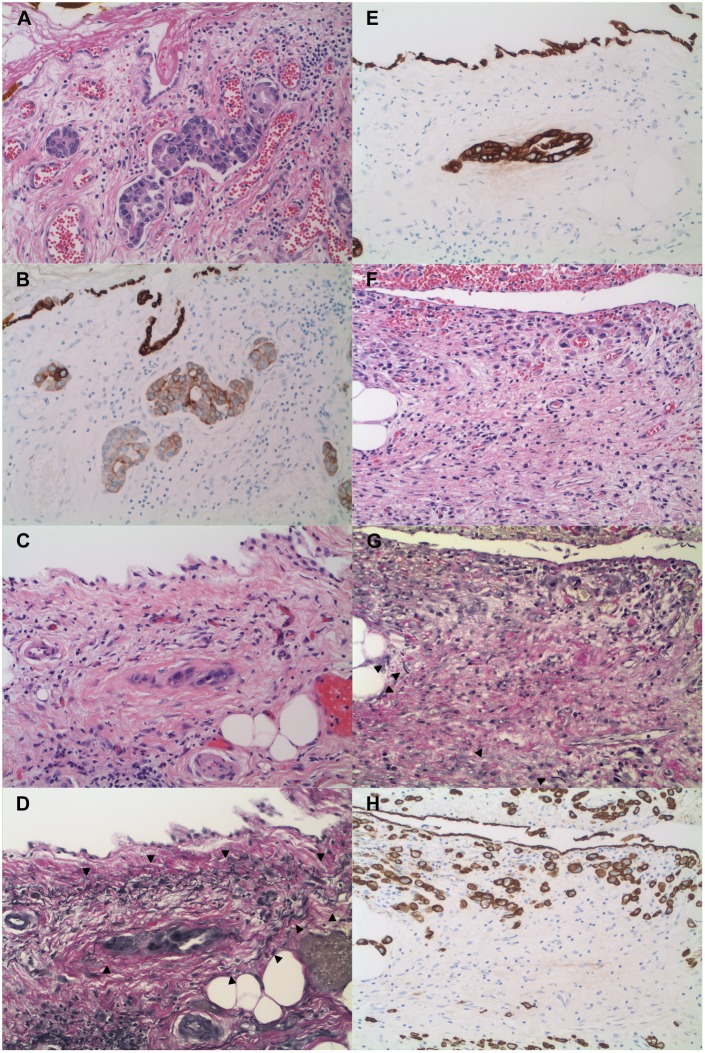
The degree of peritoneal injury correlates with the degree of stromal proliferation and cytokeratin expression, which is a reflection of the extent of tumor invasion, type of tumor, and magnitude of the tumor-associated fibroinflammatory reaction (H&E, VVG, and pan-keratin). Serosal membrane in the absence of injury, as in this adenocarcinoma of the stomach confined within lymphovascular channels subjacent to the mesothelium, lacked proliferation of peritoneal stromal cells and expression of cytokeratin (A and B, H&E and pan-keratin, 200x). This adenocarcinoma of the gallbladder focally invades the splayed elastic lamina of the peritoneum with only minimal serosal injury and mild proliferation of weakly keratin-expressing stromal cells (C-E, H&E, VVG, and pan-keratin, 200x). Poorly cohesive/signet ring cell carcinoma of the stomach extends close to the visceral serosal surface, but is associated with little inflammation, desmoplasia, and weak cytokeratin expression in a small population of peritoneal stromal cells (F-H, H&E, VVG, and pan-keratin, 200x). Arrowheads: elastic lamina.

The degree of stromal proliferation and accompanying cytokeratin expression appeared to correlate with the degree of peritoneal injury, which was a reflection of the extent of tumor invasion, type of tumor, and magnitude of the tumor-associated fibroinflammatory reaction (Fig 5). Peritoneal membranes in the absence of injury, such as control non-neoplastic tissues and peritoneum with circulating neoplastic cells in lymphovascular spaces without direct involvement of the serosal connective tissue in one case, showed neither activation or proliferation of the peritoneal stromal cell population nor its expression of cytokeratin (Fig 5A and 5B). Tumors with extensive invasion of the peritoneum, on the other hand, were typically associated with marked displacement and destruction of the elastic lamina and a prominently expanded population of keratin-expressing stromal cells. By contrast, tumors that focally invaded the peritoneum (Fig 5C and 5E), and tumors such as poorly cohesive/signet ring cell carcinoma of the stomach and adenocarcinoma ex goblet cell carcinoid of the appendix showed little tumor-associated inflammation and desmoplasia, and consequently only focal and weak keratin expression in stromal cells within or near the elastic lamina (Fig 5F–5H).

In this study, cytokeratin expression was examined using an immunostain for pan-keratin, but it could also be detected by other cytokeratin antibody cocktails such as AE1/AE3. We also examined other mesothelial or mesothelioma-associated markers such as CK7, calretinin, WT-1, D2-40, and EMA in a small number of cases, and these antibodies were less sensitive and less robust than a pan-keratin immunostain, appearing weak and in only a small subset of the keratin-expressing stromal cell population (S2 Fig). Immunostains for calretinin, and WT-1 and D2-40, in addition, also showed staining of adipose tissue and nerve fibers, and vascular endothelia, respectively, in tissues outside of the peritoneum that diminished their specificity for serosal membranes. There was no stromal cell expression of CK20. SMA did not appear to discriminate between the myofibroblastic cells of the peritoneal membrane and extraperitoneal soft tissue.

**Fig 6:**
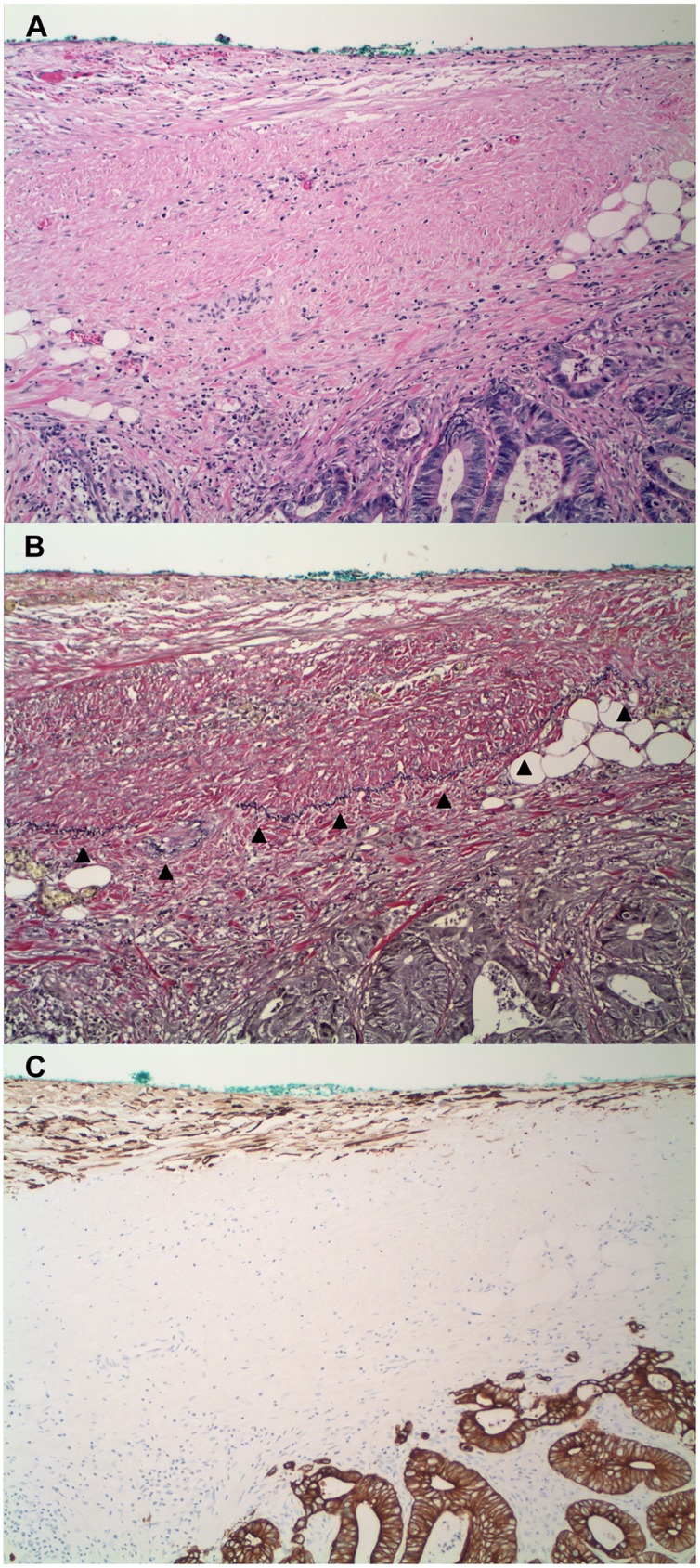
Tumors confined to the subserosa show neither invasion of the elastic lamina nor the serosal connective tissue that include the keratin-positive stromal cells of injured peritoneal membranes (H&E, VVG, and pan-keratin). Note the subserosal adipose tissue between the invasive front of the tumor and the peritoneum. (A-C, adenocarcinoma, small intestine, 100x). Arrowheads: elastic lamina.

We explored the diagnostic utility of cytokeratin immunohistochemistry in combination with a Verhoeff-Van Gieson stain in assessing tumor invasion of the peritoneal membrane. All 38 tumors without invasion of the peritoneal membrane (i.e., confined to the subserosa) showed neither invasion of the elastic lamina nor the serosal connective tissue. In cases in which there was tumor-associated peritoneal injury, there was no demonstrable tumor invasion of keratin-expression stromal cells in all cases (Fig 6). In all 82 tumors that invaded the peritoneal membrane, by contrast, the neoplastic cells invaded the elastic lamina and the connective tissue of serosal membranes with neoplastic cells that abutted or were surrounded by keratin-positive stromal cells. The association between tumors that invaded the peritoneal membrane and neoplastic cells that invaded the keratin-positive stromal cells of serosal membranes was highly statistically significant (p << 0.0001) (Table 2).

**Table 2:** Depth of tumor invasion and tumor invasion of keratin-positive stromal cells.

	Tumor without invasion of keratin-positive stromal cells	Tumor with invasion of keratin-positive stromal cells
Tumor without invasion of peritoneal membrane (i.e., confined to subserosa)	38	0
Tumor with invasion of peritoneal membrane	0	82

p << 0.0001.

## Discussion

The visceral peritoneum, composed of the surface mesothelium, its basement membrane, subjacent connective tissue, and elastic lamina, is a thin serosal membrane that invests organs of the abdomen and pelvis and their mesentery. Tumors of the gastrointestinal system that completely invade the peritoneal membrane with penetration of the visceral serosal surface are adversely associated with tumor dissemination and recurrence within the peritoneal cavity and decreased patient survival [3–8,18,19]. For tumors that partially or incompletely invade the peritoneal membrane without serosal surface involvement, however, the prognostic significance is less certain. This clinical uncertainty may be due, in part, to the under-recognition of tumor invasion of peritoneal membranes. The diagnosis requires careful evaluation and adequate sampling of the peritoneum during examination of the gross specimen [20,21]. The histopathology may be difficult to recognize due to injury and distortion of the peritoneal membrane caused by tumor invasion and associated inflammation [22,23]. Evaluation for tumor invasion of the elastic lamina can also be challenging due to anatomic variations in its composition, its displacement from the peritoneal surface, splaying, attenuation, and destruction of its elastic fibers, and variation in the technical quality of the elastic stain [10–12,21,24,25]. In fact, the elastic lamina, when invaded by tumor, can be undetected in as high as 59% of deeply invasive colorectal carcinoma, or inapparent even in tumors that penetrate the visceral peritoneal surface [9,11,12].

Gastrointestinal tumors that invade through the muscularis propria without penetration of the visceral peritoneal surface are pathologically staged as pT3 in the current AJCC and UICC classifications and cancer protocols of the College of American Pathologists [1,2]. The area of invasion by these tumors, however, broadly encompasses the subserosa just past the muscularis propria to the peritoneal stroma subjacent to the serosal surface. Deeply invasive pT3 tumors, however, exhibit more aggressive behavior than shallower tumors, and the wide range in the depth of invasion among pT3 tumors may in part account for their clinicopathologic heterogeneity and disparate clinical outcomes [5,26–28]. The visceral peritoneum is anatomically and ontologically distinct from the organ which it invests, and tumor invasion of the peritoneum may result in changes in the tumor microenvironment with altered regulation of tumor progression [12,29–31]. Therefore, stratifying pT3 tumors into those without invasion of the peritoneal membrane (i.e., limited to the subserosa) and those that invade peritoneal membranes, in a schema similar to pleural invasion by lung carcinoma [32,33], may provide an objective, anatomically defined separation of these tumors into distinct prognostic groups.

Serosal membranes are dynamic structures that, when injured by tumor invasion, undergo repair and regeneration [15,34,35]. Activated progenitor cells of serosal membranes proliferate in response to injury, which results in growth of immature spindled cells with maturation toward terminal mesothelial differentiation. In this process, the immature cells of the serosal stroma show dual keratin expression and myofibroblastic features. Keratin expression is initially weak, but gradually becomes stronger as serosal stromal cells mature toward the surface and acquire greater mesothelial cell-like features.

Expanding on earlier studies [15–17], we noted that tumor invasion of the peritoneum is accompanied by local peritoneal injury with activation and proliferation of serosal stromal cells that exhibit robust keratin expression. Keratin immunoreactivity appeared specific to the stromal cells of injured serosal membranes, forming a well-circumscribed population in or near the area of tumor invasion that typically involves almost the entire thickness of the peritoneal membrane. These cells were sharply delimited from the keratin-negative stromal cells of extraperitoneal tissue, and the area of transition between keratin-positive and keratin-negative cells correlated with the location of the elastic lamina. Tumor invasion of keratin-positive stromal cells is a specific and highly statistically significant feature of tumor invasion of peritoneal membranes, and keratin immunohistochemistry in combination with an elastic stain enhanced the detection of peritoneal invasion over an elastic stain alone in a highly sensitive and reliable manner. The combined use of keratin and elastic stains in assessing peritoneal invasion by tumor enables a more definitive diagnosis, which would in turn enhance the clinicopathologic correlation of tumor invasion with patient outcome and promote better oncologic patient care.

## Supporting information

S1 Fig Gradient of keratin expression in serosal stromal cells associated with tumor invasion (H&E, VVG, and pan-keratin). Local peritoneal injury associated with tumor invasion is characterized by activation and proliferation of serosal stromal cells that express cytokeratin. There is a gradient in the strength of keratin expression, with weaker reactivity near the elastic lamina and stronger expression toward the peritoneal surface (A-C, adenocarcinoma, colon, 20x). Arrowheads: elastic lamina.
(TIF)

S2 Fig Immunohistochemical features of peritoneal stromal cells with tumor invasion by colorectal adenocarcinoma (H&E, VVG, pan-keratin, CK7, CK20, calretinin, WT-1, D2-40, and SMA). Tumor invasion of the peritoneal membrane is characterized by activated serosal stromal cells that express pan-keratin, CK7, calretinin, WT-1, D2-40, and SMA. The degree of expression of CK7, calretinin, WT-1, and D2-40 appears less robust and seen in only a subset of the pan-keratin positive stromal cell population. There was no stromal cell expression of CK20. SMA did not appear to discriminate between the myofibroblastic cells of the peritoneum and extraperitoneal tissue. (A, H&E, 40x, arrowheads: mesothelial cells, MP: muscularis propria; B, H&E, 100x, arrowheads: mesothelial cells; C, VVG, 100x, arrowheads: splayed elastic lamina; D, pan-keratin, 100x; E, CK7, 100x, arrowheads: mesothelial cells; F, CK20, 100x; G, calretinin, 100x, arrowheads: mesothelial cells; H, WT-1, 100x; I, D2-40, 100x; J, SMA, 100x).
(TIF)

S1 Table Tumor location, tumor type, peritoneal invasion, and invasion of keratin-expressing stromal cells. (XLSX)
