# Supplementary figures and images for: Histopathology and enhanced detection of tumor invasion of peritoneal membranes
Source: PLoS One. 2017 Mar 10;12(3):e0173833. doi: 10.1371/journal.pone.0173833 (PMC5345882; doi:10.1371/journal.pone.0173833)

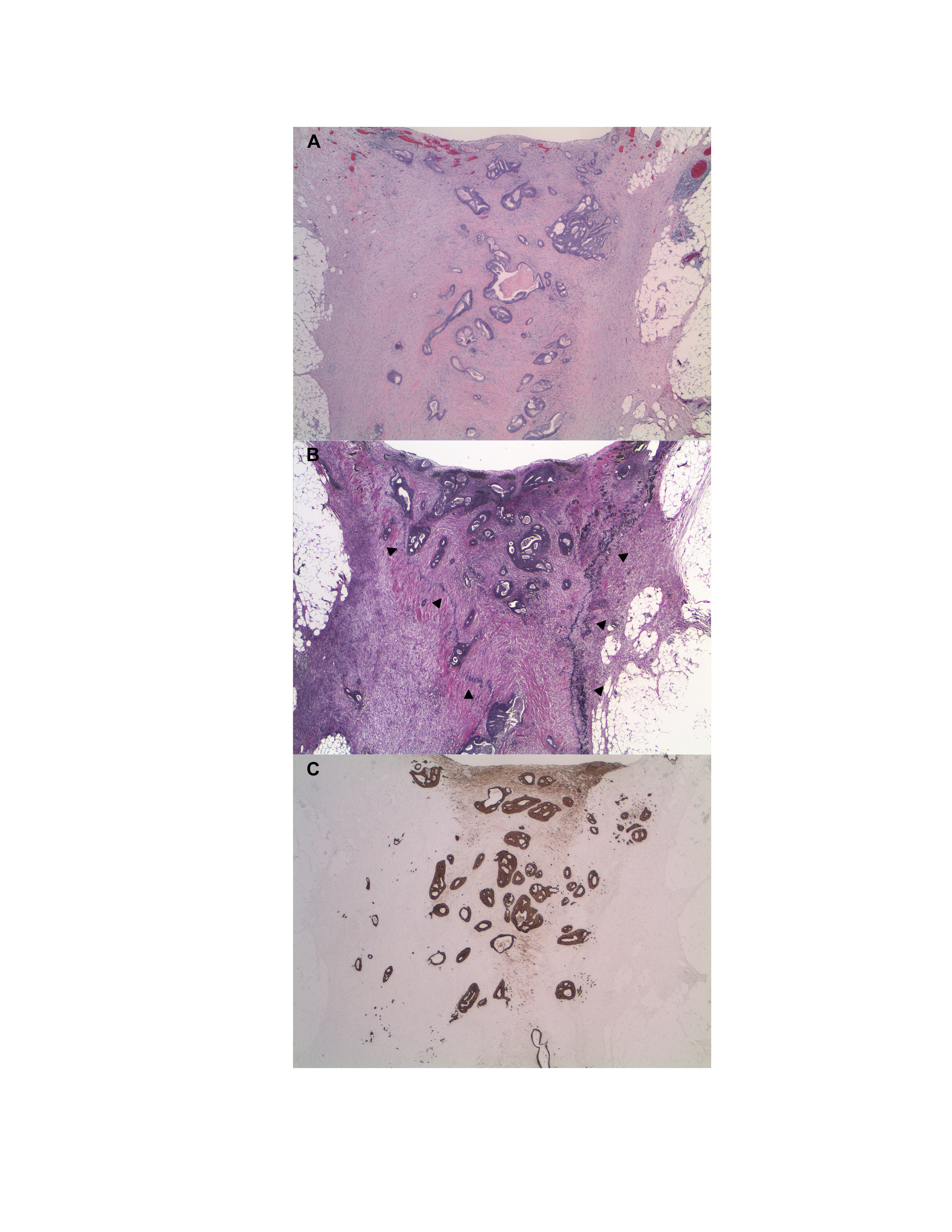

Supplement: S1 Fig — Local peritoneal injury associated with tumor invasion is characterized by activation and proliferation of serosal stromal cells that express cytokeratin. There is a gradient in the strength of keratin expression, with weaker reactivity near the elastic lamina and stronger expression toward the peritoneal surface (A-C, adenocarcinoma, colon, 20x). Arrowheads: elastic lamina. (TIF) [file pone.0173833.s001.tif]

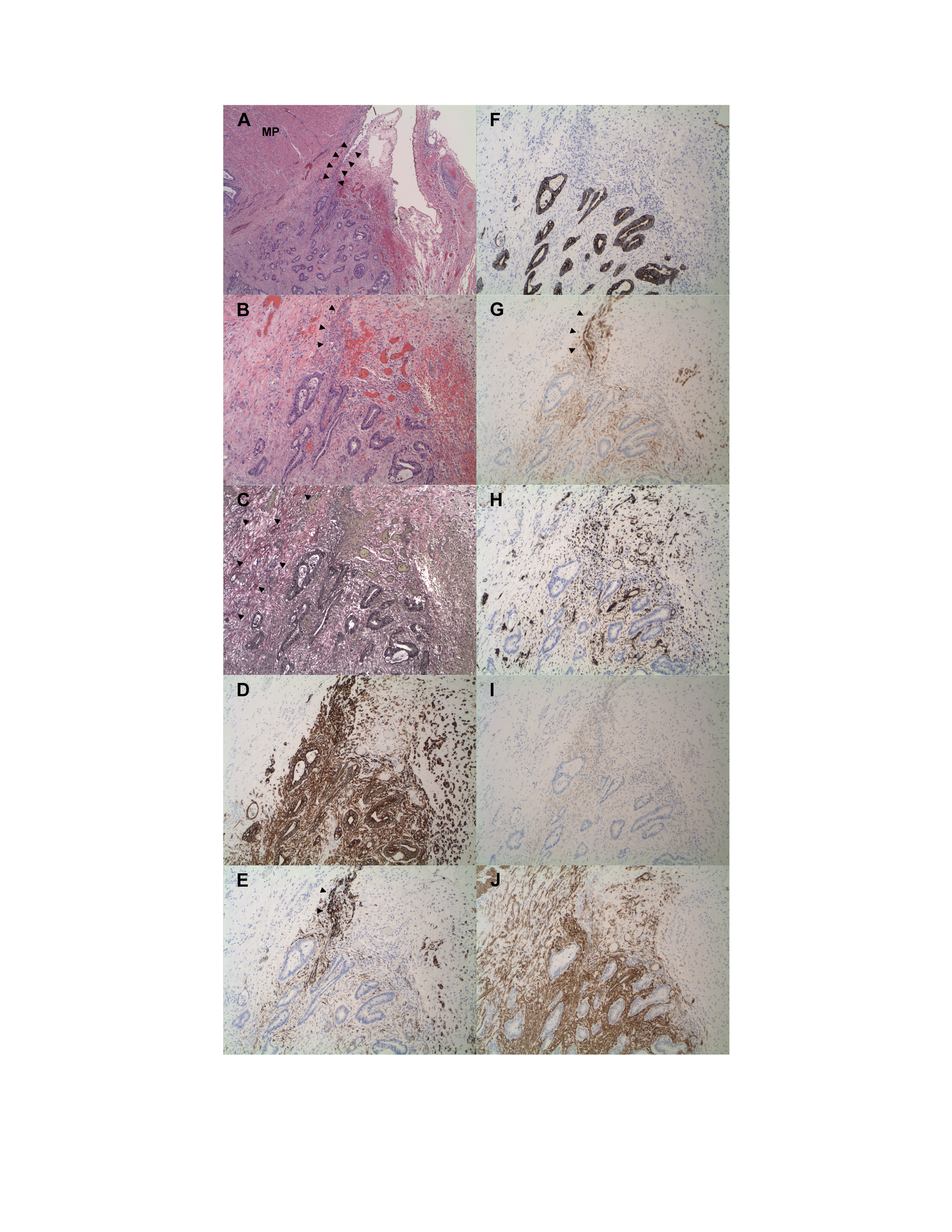

Supplement: S2 Fig — Tumor invasion of the peritoneal membrane is characterized by activated serosal stromal cells that express pan-keratin, CK7, calretinin, WT-1, D2-40, and SMA. The degree of expression of CK7, calretinin, WT-1, and D2-40 appears less robust and seen in only a subset of the pan-keratin positive stromal cell population. There was no stromal cell expression of CK20. SMA did not appear to discriminate between the myofibroblastic cells of the peritoneum and extraperitoneal tissue. (A, H&E, 40x, arrowheads: mesothelial cells, MP: muscularis propria; B, H&E, 100x, arrowheads: mesothelial cells; C, VVG, 100x, arrowheads: splayed elastic lamina; D, pan-keratin, 100x; E, CK7, 100x, arrowheads: mesothelial cells; F, CK20, 100x; G, calretinin, 100x, arrowheads: mesothelial cells; H, WT-1, 100x; I, D2-40, 100x; J, SMA, 100x). (TIF) [file pone.0173833.s002.tif]
